# Diagnostic Value Evaluation of Bed Ultrasound Compared with Wound Openness to Diagnose Tendon Rupture in Penetrating Hand Trauma at Taleghani Hospital in Kermanshah, 2019

**DOI:** 10.30476/BEAT.2021.85570

**Published:** 2021-01

**Authors:** Amirhosein Meisami

**Affiliations:** 1 Department of Emergency Medicine, Kermanshah University of Medical Sciences, Kermanshah, Iran

**Keywords:** Tendon injuries, Ultrasound, Sensitivity, Specificity.

## Abstract

**Objective:**

To determine the sensitivity, specificity and accuracy of ultrasound to diagnose the patients with tendon rupture of upper extremity referred to Taleghani Hospital’s center of Kermanshah in 2019.

**Methods:**

This was a diagnostic value study which performed on 113 patients with non-fracture penetrating hand trauma. In the first stage, all patients have been diagnosed with tendon injury by a first-year resident and then ultrasound was performed by a trained 2^nd^ year resident in emergency medicine ward and the results were recorded in a checklist. Further examination of the tendon was performed as well as exploring the site for the patients after the patient was transferred to the orthopaedic service. Final result was recorded in the checklist. Data were analysed by SPSS software and sensitivity and specificity of ultrasound have been calculated.

**Results:**

Results showed that ultrasound was able to identify 73 patients of 77 individuals with tendon injury. Of the patients with complete rupture, 45 individuals were correctly diagnosed based on the results of surgery in ultrasound test. Ultrasound and surgical findings were significantly different. Of the 36 individuals without confirmed rupture in surgery, 10 cases were diagnosed with tendon rupture by ultrasound. Of 52 cases of complete rupture based on surgical findings, 45 individuals were correctly diagnosed based on the results of surgery. Twenty-one patients were correctly diagnosed based on ultrasound out of 25 cases of partial rupture based on surgical findings.

**Conclusion:**

Overall, the results of the present study show that ultrasound is not very sensitive and specific in diagnosing of upper extremity tendon rupture and cannot be used as a reliable alternative in diagnosing of upper exteremity rupture; however, further studies is essential according to the limitations of this research. The limitations were low sample size in subgroups analysis based on the presence of complete or partial rupture and performing the ultrasound by an emergency medicine resident who is less experienced rather than radiologists.

## Introduction

Tendon ruptures are common musculoskeletal injuries occurring along with penetrating trauma worldwide. These injuries are one of the most common musculoskeletal complaints responsible for 7% of all referrals to doctors in the United States [1]. Approximately 30% of emergency referrals is a common injury among factory workers, housewives, and athletes include hand and finger injuries; of which 30% are tendon lesions [2, 3]. Therefore, magnetic resonance imaging (MRI) has been cited as the choice method to diagnose tendon injuries in the American College of Radiology report. However, this method is costly and inaccessible under acute conditions [4]. Advances in ultrasound technology have shown that this technique is more suitable than alternative and complementary tool for diagnosing tendon injury compared to MRI [4]. Although wound exploration is known as a traditional and accurate method to examine tendon rupture and soft tissue injuries, ultrasound could be used to assess traumatic tendon, ligament, and peripheral nerves’ injuries. In a study, ultrasound sensitivity, specificity and accuracy was estimated as 98%, 100%, 99%, respectively [5]. Notably, Tendon / ligament ruptures may be complete or partial. Ligament elongation has shown as an increasing diffuse ligament thickness with no obvious rupture that is indicated in ultrasound. However, the rupture is seen as a hypovaccus area within the thickened ligament. In complete ruptures, the fibrillar pattern is completely cut off and the fibers are widely distributed. Partial ruptures are more difficult to detect which could be seen as linear hypovaccus areas perpendicular to the longitudinal axis of tendon / ligament fibres, and also fluid collection or hematoma may be seen at the site of rupture. The locations of the rupture and the damaged fibres can be demonstrated by ultrasound which is used in reconstructive surgery plan [6]. Although computed tomography (CT) scans or MRI findings can also be used to diagnose ligament rupture, ultrasound can assess trauma dynamic site which is a low invasive and non- traumatic method. Another study has shown that ultrasound has MRI value to detect rupture in the tendon. Ultrasound can also diagnose other causes such as fingers flexor tendon tenosynovitis and arthritis [6]. Furthermore, ultrasound can be considered as a non-invasive and appropriate diagnostic tool to identify patient’s plan in people with no desire to perform surgical exploration. Since few studies have investigated the diagnostic value of ultrasound, in this study, we purpose to evaluate the diagnostic value of ultrasound in bed for patients with penetrating hand trauma.

## Materials and Methods

The present study was a functional diagnostic study. The sample size was determined in terms of the study by Mohamad-Rezaei *et al*., [7] 

$\mathrm{nse}=\frac{{z^{2}}_{\frac{3}{2}}\overset{\text{\textasciicircum{}}}{\mathrm{se}}\left(1-\mathrm{se}\right)}{d^{2}\times \mathrm{prev}}$

$\mathrm{nsp}=\frac{{z^{2}}_{\frac{3}{2}}\overset{\text{\textasciicircum{}}}{\mathrm{sp}}\left(1-\mathrm{sp}\right)}{d^{2}\times (1-\mathrm{prev})}$

Based on the statistical population, 113 individuals with penetrating hand trauma have been enrolled in the study who referred to Taleghani Hospital Emergency ward of Kermanshah from March 22, 2018 to March 20, 2019. Inclusion criteria were penetrating trauma without fracture.

All patients have been diagnosed with tendon injury by the first-year resident of emergency medicine at the first stage. Afterwards, the ultrasound was performed by the trained second-year resident of emergency medicine in the emergency department and the results were recorded in an out-of-file form. Ultrasound was performed using the Toshiba device at the time of admission. Following these steps, the patients was transferred to the orthopaedic services and the supplemental checking were also performed to examine and explore tendon site for the patients. At this stage, the patient’s outcome was recorded in a separate form and kept in the patient’s file. The results of ultrasound and orthopaedic evaluation were recorded in researcher’s and patients’ files after its completion in the operating room. The initial ultrasound results were compared with the gold standard method (orthopaedic exploration). Additionally, ultrasound sensitivity and specificity were determined. The final data were analysed by using a descriptive statistic (frequencies, percentage, mean, kappa test). The statistical significance level of this study was considered as 0.05.

## Results

**Fig. 1:**
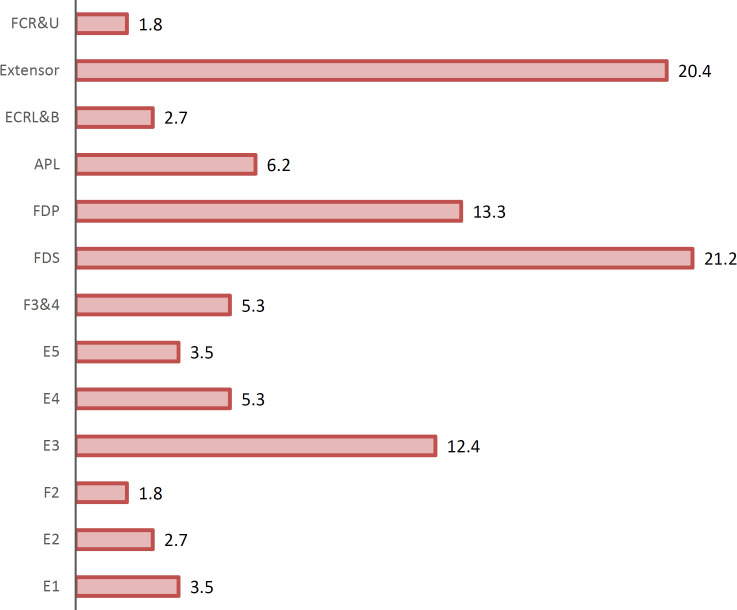
Frequency (Percentage) of studied patients by type of examined tendon. FCR&U,Flexor Carpi Radialis & Ulnaris ; Extensor; ECRL&B, Extensor Carpi Radialis Longus & Brevis; APL, Abductor Pollicis Longus FDP, Flexor Digitorum Profundus; FDS,Flexor Digitorum Superficialist ; F3&4,Flexor 3،4;E5, Extensor5

This study was performed on 113 patients with penetrating tendon injuries. The mean age of patients was 31±7.36 years old. Table 1 shows the patients’ gender and age groups frequency distribution. Approximately 87% of patients were men and 13% were women. Moreover, the majority of the included patients (54.9%) were in the age range of 31-40 years old. In this study, 63 individuals (55.8%) had left-sided trauma and 50 individuals (44.2%) had right-sided trauma. The frequency distribution of the damaged tendon is shown in Figure 1. The results show that Flexor digitorum superficialis tendon with 24 patients (21.2%) was the most common type of the injured tendon. According to the results of surgical exploration, there was 52 patients with complete rupture, 25 patients with partial rupture, and 36 patients with no rupture. Furthermore, there was 45 patients with complete rupture, 38 patients with partial rupture, and 30 patients with no rupture according to the ultrasound results.

**Table 1:** Frequency distribution of under study patients by gender and age groups

**Frequency**	**Percentage**	
		Gender
98	86.7	Male
15	13.3	Female
Age group		
6	5.3	Less than 20 years
41	36.3	21-30 years old
62	54.9	31-40 years old
4	3.5	More than 40 years

Kappa coefficient was used to compare the diagnostic values of ultrasound and surgery (Table 2).

**Table 2:** Comparison of surgical findings and ultrasound findings in terms of Complete Rupture

**Surgical Findings**				
**Complete Rupture**	**Without Rupture**	**Total**		
45	10	55	Complete Rupture	Ultrasound Findings
7	26	33	Without Rupture	
	52	36	Total	

Table 3 represents the ultrasound diagnostic value estimations in diagnosis of complete tendon rupture. As shown, the ultrasound sensitivity is 86.5% and its specificity is 72.2% f in detecting complete tendon rupture. Additionally, the positive and negative predictive value of ultrasound is 81.8% and 78.9%, respectively. Table 4 shows the ultrasound diagnostic values and surgery in diagnosis of rupture and without rupture and kappa coefficient was 71% which indicating that there is a medium agreement between these two diagnostic methods [8]. In Table 5, the estimations of the diagnostic value of ultrasound are presented in detecting the presence of partial tendon rupture. As shown, the ultrasound sensitivity and specificity is 94.8% ​​and 72.2%, respectively in detecting the presence of partial tendon. Moreover, ultrasound positive and negative predictive value is 87.9% and 86.7%, respectively in detecting the presence of partial tendon.

**Table 3:** Estimations of ultrasound diagnostic value in detecting of complete tendon rupture

**Estimations **	**Confidence interval**	
86.5	94.4– 74.2	Sensitivity
72.2	8.85 – 54.8	Specificity
81.8	88.5 – 72.4	Positive predictive value
78.9	88.4 –64.4	Negative predictive value
80.1	88.3 – 70.9	Accuracy

**Table 4:** Comparison of surgical findings and ultrasound findings in terms of Rupture (Complete or Partial)

**Surgical Findings**				
**With Rupture**	**Without Rupture**	**Total**		
73	10	83	With Rupture	Ultrasound Findings
4	26	30	Without Rupture	
77	36		Total	

**Table 5:** Estimates of ultrasound diagnostic value in detecting tendon rupture (complete or partial)

**Estimation**	**Confidence interval**	
94.8	98.6 – 87.2	Sensitivity
72.2	85.8–54.8	Specificity
87.9	92.5– 81.1	Positive predictive value
86.7	5.94 –72.0	Negative predictive value
87.6	1.93 – 80.1	Accuracy

## Discussion

The fingers traumatic tendon rupture is a common finding in soft tissue injury of musculoskeletal trauma. In this regard, surgery is one of the alternative treatment options to treat tendon ruptures in the fingers; however, post-operative complications like adhesion around the tendon can be observed by using this method and even experienced surgeons in treated patients. In the majority of patients, rupture or injury diagnosis of the finger’s tendon can be diagnosed by a history of trauma or clinical examination but CT scan findings are useful in cases when flexor tendon rupture cannot be diagnosed [8, 9]. Using ultrasound technology in tendon rupture diagnosis is mainly limited to large tendons such as Biceps, Quadriceps, and Achilles, and ultrasound findings can provide additional findings to confirm the clinical diagnosis of tendon injury [10, 11]. The ultrasound and surgery diagnostic values show that kappa coefficient was 60% in diagnosing the complete rupture and without rupture, which is indicating that there is a medium agreement between these two diagnostic methods [12].

Notably, there are limited findings on the efficacy of ultrasound in the diagnosis of tendon trauma in the fingers [10].

In this study, 113 patients were studied with penetrating hand tendon injury. Of them, 86.7% were men and the mean age was 31 years old. In the study by Lee *et al*., [13] performed in the United States, using the ultrasound evaluation of flexor tendon laceration were evaluated in 10 patients, 10 hands, and 20 traumatic’s finger flexor tendons. Jeyapalan *et al*., [14] have been studied 17 patients and 18 fingers with trauma in the United States. Moreover, Shakarchi *et al*., [15] were studied an ultrasound accuracy in 20 patients with mean age of 38.6 years old to diagnose ulnar collateral ligament thumb injury which of them 60% were men. 

A study [16] in Netherlands performed on 21 patients with rheumatoid arthritis with a mean age of 61 years old in comparing between ultrasound and MRI results to diagnose of finger’s tendon extensor partial rupture. In Wu *et al*., [17] study which is performed in the United States, 34 patients were included in the study and the ultrasound was done on the tendon injury of the patients. In consequence, this study found 6 fingers and 11 hands with tendon injury. Zhang *et al*., [18] in China evaluated an ultrasound value with high frequency in diagnosis and surgical repair of finger’s traumatic tendon rupture. Correspondingly, 92 patients (185 fingers) with trauma have been evaluated and observed that the patients’ mean age was 32.6 years old and 59% of them were men. 

Ravnic *et al*., [19] in the United States was performed ultrasound in diagnosis and evaluation of the flexor tendon injury location and 81 corps were also evaluated. According to previous studies [13, 15, 18], it seems that the number of the samples in the present study is comparable to other studies, and the gender and age of the patients indicated that occupational trauma was the main cause of this injury.

The present study found that flexor digitorum superficialis (FDS) and flexor digitorum profundus (FDP) were the most common traumatic tendons and resembling with other studies such as Bianchi *et al*., [20] and Clavero *et al*., [21]. These studies resemble that FDP injury is the most common form of flexor tendons closure injury; and FDP and FDS injuries can also be as an active finger flexion due to patio hyper extension injury. Notably, this injury is predominantly among young men participating in contact sports like Rugby Football.

Therefore, these tendons were most likely to have trauma according to the fact that most of the traumatic injuries are the hand volar surface and FDP and FDS tendons have the highest contact area. In the present study, we found that ultrasound is able to fully detect all individuals with complete tendon rupture to detect sensitivity, specificity, positive and negative predictive values as 100%. However, this diagnosis was different in incomplete rupture. It was also observed that sensitivity, specificity, positive predictive value and negative predictive value was 94.8%, 72.2%, 87.9% and 86.7%, respectively in the diagnosis of incomplete rupture. 

In a study of Lee *et al*., [13] 20 tendons, 12 healthy tendons, 2 partial laceration tendons, and 6 complete laceration tendons were detected. out of which, 6 tendons were with complete rupture, 5 individuals were diagnosed by Ultrasound, and out of 12 healthy tendons (6 patients), 2 tendons were mistakenly reported by ultrasound as positive. 

Jeyapalan *et al*., [14] study observed that 4 patients (23.52%) out of 17 had normal tendon of finger’s tendon trauma. In 3 patients (17.64%) completed rupture, 5 patients (29.42%) partial rupture, 5 patients (29.42%) increasing thickness, fibrosis and the decreased tendon motion were observed.

Complete rupture was observed in 3 patients. Surgical and exploration procedures were performed for these patients whereas in 2 patients (66.66%) an obvious rupture was observed. The results analysis showed that ultrasound could prevent the additional exploration of traumatic patients. Shekarchi *et al*., [15] found that 7 patients (35%) were diagnosed by ultrasound and 7 patients (35%) with ligament rupture by MRI among patients with thumb trauma. In this study, sensitivity, specificity, positive predictive value and negative predictive value was observed as 71.42%, 84.61%, 71.42% and 84.61%, respectively in diagnosis of ligament rupture by ultrasound. In another study, Al-Hourani [22] claimed that ultrasound had a sensitivity (75%), specificity (99%), positive predictive value (99%), and negative predictive value (50%) in diagnosis of hand tendon rupture. The results of the study by Swen *et al*., [16] showed that sensitivity and specificity of ultrasound were 67% and 100% in diagnosis of complete hand tendon rupture. Moreover, for the diagnosis of incomplete ultrasound of rupture, sensitivity, specificity, positive and negative predictive values were 33%, 89%, 50%, and 75%, respectively. 

Wu *et al*., [17] showed that ultrasound sensitivity and specificity were 100% and 95% in diagnosis of tendon injury of upper extremity in patients with trauma. Also, clinical examination was 100% and 76%, respectively. Zhang *et al*., [18] showed that preoperative ultrasound provides useful information to identify the location and type of tendon rupture and surgery with an ultrasound guide that could decrease the extent of tendon adhesion after surgery. In the study of Ravnic *et al*., [19] it was reported that accurate diagnosis of flexor tendon injury was observed in 96.2% of patients. Finding the exact location of rupture was also reported in 78% of the patients. Small finger injury was the most difficult to detect in 66.7% of cases. Soni and colleagues [23] also represent that high-resolution ultrasound is the most accurate method for clinical examination and MRI to diagnose extensor tendon injuries because the diagnosis of rupture with clinical examination and MRI is difficult in complete and partial injuries. The evaluation of the present study compared with other studies showed that the complete rupture diagnosis was very favourable and the rate of incomplete rupture showed better results rather than other studies. The report and expression of the above studies indicate the comparison of differences and similarities between the present and their study.

In conclusion, the present study show that ultrasound does not have high sensitivity and specificity in diagnosis of partial tendon rupture. Therefore, it cannot be used as a reliable alternative method for diagnosis of upper extremity partial rupture. However, further studies are necessary in this field due to the limitations of this study especially low sample size in subgroups’ analysis based on the presence of complete or partial rupture and performing ultrasound by an emergency medicine resident who was less experienced than radiologists. 

Study Limitations and Suggestions

● The results of clinical examinations were not evaluated.

● Other imaging tools including CT scans and MRI were not evaluated in this study.

● It is recommended that further studies must be conducted in this field and more patients should be evaluated.

● It is suggested that another study should be performed in which patients will be divided into multiple groups and other imaging tools will be tested.

● It is suggested that lower extremities should also be considered in the future studies.

● It is suggested that ultrasound must be performed by both emergency medicine physicians and radiologists to assess the degree of agreement between them in the future studies.
